# A multimodal dataset for socially aware navigation of heavy-duty construction robots

**DOI:** 10.3389/frobt.2026.1873168

**Published:** 2026-07-17

**Authors:** Carlos A. P. Pizzino, Phani-Teja Singamaneni, Micael S. Couceiro, Rachid Alami

**Affiliations:** 1 Ingeniarius, Lda., Alfena, Portugal; 2 LAAS-CNRS, Universite de Toulouse, Toulouse, France

**Keywords:** autonomous mobile robot, benchmarking and evaluation, construction robotics, human-aware path planning, human-robot interaction, multimodal robotics, socially aware navigation

## 1 Introduction

Socially aware navigation has become a central requirement for mobile robots operating in human-populated environments (Singamaneni et al., 2024), yet the datasets currently available to the community remain largely centred on indoor platforms, teleoperated systems, or simplified human-motion traces. This creates a significant gap for field robotics applications in which robots are larger, heavier, operate in less structured outdoor environments, and must interact with workers under stricter safety constraints. In particular, heavy-duty construction robots introduce challenges that are still poorly represented in public datasets, including large robot footprints, reduced manoeuvrability, multimodal sensing under outdoor conditions, corridor-like shared passages, and the need for transparent human-robot interaction (HRI) beyond pure obstacle avoidance (Couceiro et al., 2024).

This Data Report addresses that gap by presenting a multimodal dataset for socially aware navigation and voice-based HRI collected in the context of HARD-HAT, a project funded through the euROBIN EU Project^1^. The dataset was acquired using a retrofitted ROS-enabled Bobcat compact track loader operating in a construction-like testbed (Portugal et al., 2021), while executing repeated human-shared navigation trials and complementary rule-based natural language understanding (NLU) tests under controlled and noisy conditions.

The dataset is designed to support research across different dimensions of social navigation. On the one hand, it provides a reproducible benchmark for comparing geometric navigation against CoHAN-based socially aware navigation on a heavy robotic platform using a common sensing, localisation, and control stack (Singamaneni et al., 2021). On the other hand, it extends the notion of social navigation to a practical interaction layer through a subsidiary voice-command pipeline, including audio capture, speech-to-text outputs, lexical normalisation, rule-based intent extraction, and ROS-level event traces (Certo et al., 2025).

While the navigation subset demonstrates consistent socially aware behaviour across repeated trials, the voice-interaction subset includes both successful and failure cases collected under realistic construction-like acoustic conditions. In particular, command-recognition performance decreases substantially with operator distance, providing a representative benchmark of the challenges associated with speech interaction around heavy-duty robotic machinery.

This article therefore focuses on describing the dataset itself: how it was collected, how it was curated, what modalities and metadata it contains, and how it may be interpreted and reused. The aim is to make available a structured and traceable resource for the community, enabling future work on socially aware navigation, HRI, and safety-critical autonomy in construction and other demanding outdoor domains.

## 2 Methods used to collect the data

This section describes the methodology adopted to acquire, organise, and curate the dataset, covering the experimental environments, robotic platform, sensing and software architecture, data collection protocols, and the main preprocessing steps applied before packaging. Given the intended role of the dataset as a reusable benchmark for socially aware navigation and voice-based HRI in heavy-duty field robotics, particular attention is devoted to reproducibility, traceability, and practical reuse.

### 2.1 Experimental environments

Data was collected in two complementary environments selected to balance realism, repeatability, and safety. The primary environment was an outdoor construction-like testbed of approximately 1000 $m^{2}$ , used to reproduce representative heavy-duty operation conditions, such as uneven terrain, dust, open-space acoustics, and the presence of static and dynamic obstacles. Within this testbed, the navigation benchmark focused on a corridor-like shared passage scenario, representative of common worksite situations in which workers and heavy machinery must negotiate space in constrained areas. A secondary environment was an indoor garage area, used mainly for the voice-interaction and rule-based NLU trials under more controlled acoustic conditions. This indoor space enabled the collection of baseline recordings with reduced wind and environmental variability, thereby supporting comparison between quiet conditions and more challenging outdoor operation.

### 2.2 Robotic platform and sensors

**FIGURE 1:**
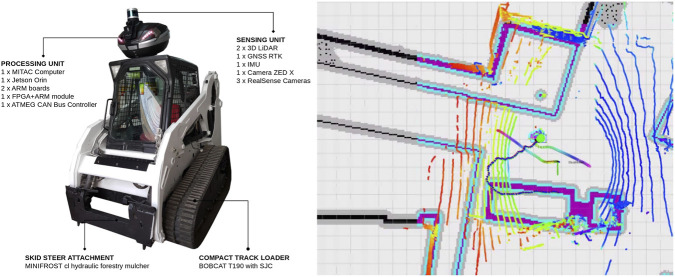
Robotic platform and sensing overview. Left: retrofitted Bobcat T190 compact track loader used for data collection, with the main sensing elements indicated. Right: representative RViz visualisation of the multimodal data streams available in the dataset, including localisation, perception, human tracking, and navigation-related information.

The dataset was collected using a retrofitted Bobcat T190 compact track loader, representative of a heavy-duty unmanned ground vehicle for construction-oriented transport tasks (Couceiro et al., 2024). The platform, presented in Figure 1, was instrumented to support autonomous navigation, human-aware operation, and multimodal data logging in realistic outdoor conditions. Its use in this dataset is particularly relevant because, unlike lightweight indoor robots, it introduces the constraints typical of heavy machinery, such as larger footprint, reduced agility, and stricter safety requirements in human-shared environments.

The sensing suite combines complementary spatial, visual, and proprioceptive modalities. It includes a 3D LiDAR for environment perception and obstacle representation (LeiShen LIDAR C16), a stereo camera for visual perception and human tracking support (Stereolabs ZED-X), GNSS-RTK for outdoor positioning (Emlid GNSS Reach RS2), and an IMU for motion estimation and pose stabilisation (Rion AH-200C). For the voice-interaction subset, the robot was also equipped with a directional microphone (RØDE VideoMic GO II) integrated with the onboard computer to capture operator speech in both controlled and noisy conditions, supporting the speech-to-text and rule-based NLU pipeline.

In parallel, internal robot-state and control data are logged through a ROS-CAN software bridge to the vehicle, allowing planner decisions, localisation outputs, and commanded motion to be recorded consistently with the sensor streams (Portugal et al., 2021). Together, the robotic platform and sensor configuration provide a multimodal basis for studying socially aware navigation and HRI in large-scale outdoor robots.

### 2.3 System architecture and software stack

The dataset was generated from a ROS-based autonomy stack deployed on the robotic loader and organised around four main layers: perception and localisation, human tracking, navigation and control, and interaction/logging. The software stack runs on ROS Noetic and combines standard navigation components with project-specific integrations.

#### 2.3.1 Perception and localisation

The perception and localisation subsystems constitute a layer of the autonomous navigation pipeline, enabling robust state estimation and environment understanding in dynamic, construction-like environments. Accurate localisation is achieved through the integration of LiDAR, IMU, and GNSS data using a tightly-coupled sensor fusion framework LIO-SAM Shan et al. (2020), which provides locally consistent odometry through LiDAR scan matching and IMU pre-integration, globally consistent positioning via GNSS corrections, and drift mitigation through loop closure and sensor fusion.

#### 2.3.2 Human tracking

Human detection is performed using the stereo vision pipeline, where the ZED SDK provides real-time object detection and tracking. To interface with the navigation stack, a dedicated ROS node converts raw detections into a simplified representation based on pedestrian centroids. This abstraction reduces computational complexity while preserving the spatial information required for human-aware planning. This representation is subsequently published in a format compatible with the human-aware navigation framework, allowing modules to model pedestrians as dynamic agents.

#### 2.3.3 Navigation and control

At the navigation level, two configurations were considered. The baseline uses a conventional TEB-based local planner (Rösmann et al., 2012), while the socially aware configuration combines the same navigation backbone with the CoHAN 2.0 package^2^, implemented through HATEB (Singamaneni and Alami, 2020) and the associated human-aware costmap layers (Singamaneni et al., 2021). In this way, the comparison remains controlled: the global navigation pipeline, robot constraints, and sensing inputs are kept constant, while the local planning behaviour is augmented with social-awareness mechanisms. Human state information is provided by a dedicated tracking module, which publishes pedestrian position and related information to the ROS graph for use both in online planning and in offline evaluation.

#### 2.3.4 Interaction and logging

For the interaction subset, the system integrates an audio processing chain, speech-to-text output, and the nlu_rule_based package^3^, which maps recognised utterances to a restricted set of robot-relevant intents. These commands are not sent directly to the vehicle actuators, but pass through the same supervisory and safety logic used by the autonomy stack. In parallel, all relevant streams are recorded through ROS bagging, including robot state, perception topics, planner outputs, human-tracking data, and audio/NLU-related events, ensuring full traceability of each trial.

### 2.4 Data collection protocol and period

The data collection process followed a controlled and repeatable protocol designed to ensure fair comparison between navigation configurations while maintaining safe operation of the robotic platform. For the navigation subset, repeated tests were carried out in a corridor-like outdoor scenario, where the robotic loader autonomously navigated between fixed start and target locations while interacting with a pedestrian followed a predefined head-on interaction trajectory designed to create a repeatable human–robot encounter. In each trial, the pedestrian walked towards the corridor centreline at an approximately constant speed until reaching a predefined interaction point. Upon reaching this location, the pedestrian stopped and remained stationary for the remainder of the interaction, allowing the robot to autonomously decide how to negotiate the shared space and complete its navigation task. The pedestrian behaviour remained identical across all trials and planner configurations, ensuring that any observed differences in robot behaviour could be attributed solely to the navigation strategy under evaluation rather than variations in human motion.

Trials were executed under the same vehicle constraints and sensing configuration, alternating only between the baseline planner and the socially aware CoHAN-based configuration.

The data collection protocol captures a multi-layer representation of the system, including:

- Perception Data: Stereo camera streams, LiDAR point clouds, Detected pedestrian positions and tracking data;
- Localisation Data: LiDAR-inertial odometry, GNSS-RTK positioning, IMU measurements, TF transformations;
- Planning and Control Data: Global paths, Local trajectories for each planner, Predicted robot and agent future states;
- Interaction and Evaluation Data Robot–pedestrian distance metrics;
- Close-contact event indicators, and
- Collision detection signals.

For the voice-interaction subset, the protocol combined indoor baseline recordings with outdoor trials under construction-like noise. Controlled audio playback tests were used to ensure repeatability, while live operator tests captured more realistic interaction conditions at different distances from the robot.

Across both subsets, all trials were recorded through ROS logging, with metadata describing the active configuration, environment, and test condition. All data streams are synchronised and recorded in ROS bag files, ensuring a consistent temporal and spatial reference across modalities. Following data collection, a structured post-processing pipeline can be applied.

### 2.5 Filters, preprocessing, and curation

Only light preprocessing was applied before dataset curation, with the goal of preserving the original sensor and system outputs while improving consistency and reuse. For the navigation subset, ROS logs were checked for completeness, synchronised through their timestamps, and organised together with the corresponding metadata describing planner configuration, trial condition, and scenario. No filtering was applied to alter the planner behaviour retrospectively; the dataset therefore reflects the system as executed during the experiments.

For the voice-interaction subset, audio streams were preprocessed to support speech recognition in noisy conditions. This included high-pass filtering to attenuate low-frequency engine noise, followed by noise reduction using spectral gating. Both stationary and non-stationary noise-reduction modes were considered depending on the recording condition. Audio was then resampled to a format suitable for the speech-to-text pipeline. In parallel, transcripts and NLU outputs were stored with the corresponding trial metadata.

During curation, runs with incomplete logs or invalid metadata were excluded, and the final dataset was organised into consistent subsets for navigation and voice interaction. The public release includes the curated logs, metadata, and supporting documentation needed to interpret and reuse the data.

### 2.6 Dataset structure, metadata, and repository

The dataset (Pizzino and Couceiro, 2026) is organised into two main subsets: *navigation* and *voice interaction*. Each run is stored as an individual data unit together with the minimum metadata required for interpretation and reuse. The repository links for the different subsets of the dataset are summarised in Table 1.

**TABLE 1:** Repository links for the main dataset subsets.

Subset	Content	Link
Socially aware navigation	CoHAN-based planner trials	CoHAN
	TEB local planner trials	TEB
Voice interaction	Audio, transcripts, and NLU outputs	NLU
Documentation	Metadata and reuse notes	README

The dataset is primarily intended for benchmarking socially aware navigation and voice-based interaction on heavy-duty outdoor robots in construction-like settings; users should note that it reflects the sensing, actuation, and safety constraints of a specific robotic platform and testbed.

#### 2.6.1 Dataset replay and reproducibility

The dataset is distributed as ROS bag recordings together with metadata files and supporting documentation intended to facilitate replay, inspection, and benchmarking. All data were collected using ROS Noetic on Ubuntu 20.04, and the recorded topics can be replayed using the standard ROS bag infrastructure.

The navigation subset was generated using a ROS-based autonomy stack composed of perception, localisation, human tracking, navigation, and logging modules. Core software dependencies include move_base, global_planner, teb_local_planner, HATebLocalPlannerROS, CoHAN 2.0, LIO-SAM, and the ZED ROS Wrapper for human detection and tracking.

## 3 Data analysis and validation

To support reuse of the dataset, this section provides a concise analysis of its content and a set of technical validation checks on the recorded data. The goal is not to present the results of a specific scientific study, but rather to characterise the dataset in terms of coverage, consistency, and practical value for benchmarking socially aware navigation and voice-based HRI. The following subsections therefore summarise the main statistics of the collected data and assess their suitability for future comparative studies, replication efforts, and downstream analysis.

### 3.1 Dataset summary statistics

The current release combines two subsets: a *navigation* subset and a *voice-based HRI* subset. Table 2 summarises the main characteristics of the collected data. Together, these subsets provide multimodal logs suitable for benchmarking socially aware navigation, analysing HRI, and studying command comprehension under realistic field conditions.

**TABLE 2:** Summary of the main characteristics of the released dataset.

Subset	Main content	Summary
Navigation	Robot state, localisation, perception, planner outputs, human-tracking data, and online benchmarking topics	10 test sessions with human presence and interaction, including 10 paired trials comparing TEB and the CoHAN-based configuration in a corridor-like scenario
Voice-based HRI	Audio capture, speech-to-text outputs, and rule-based NLU interpretations	Audio playback tests with 3 speakers and 5 commands per speaker, complemented by live operator trials at different distances from the robot under controlled and noisy conditions

The navigation subset contains 20 complete traversals, comprising 10 runs with the TEB planner and 10 runs with the CoHAN-based configuration. For each run, an effective assessment window was identified corresponding to the period during which meaningful human–robot interaction occurred. The start and end times of these windows were determined from the recorded trajectories and used for the computation of all interaction metrics. Across all trials, the effective interaction windows ranged from 29.5 s to 43.4 s. The mean interaction duration was 31.0 s for the TEB configuration and 40.6 s for the CoHAN-based configuration. The recorded experiments span approximately 20.1 min for the TEB trials and 23.5 min for the CoHAN-based trials, corresponding to a total recorded operation time of 43.6 min. Within these recordings, the cumulative usable interaction time was 310.3 s (5.2 min) for TEB and 405.5 s (6.8 min) for the CoHAN-based configuration, yielding a total of 715.9 s (11.9 min) of benchmarked human-robot interaction data across the dataset. These interaction windows represent the portion of each recording used for benchmarking and correspond to the periods in which the pedestrian and robot were simultaneously engaged in the evaluated navigation task.

### 3.2 Technical validation

Technical validation was carried out to ensure that the released data are complete, internally consistent, and suitable for reuse. The main checks were as follows:

- Log completeness: each retained run was verified for the availability of the core data streams required for interpretation and replay.
- Time consistency: synchronisation between modalities was verified through recorded timestamps and TF information.
- Metadata consistency: each run was cross-checked against environment, and active system configuration.
- Navigation subset validation: runs were verified to contain the required robot-state, localisation, perception, planner, human-tracking, and evaluation topics.
- Voice subset validation: runs were verified to contain the corresponding audio record, transcript output, NLU interpretation, and test-condition metadata.
- Curation rule: runs with incomplete logs or inconsistent metadata were excluded from the released dataset.

Beyond these checks, the dataset was also verified to cover the intended experimental variability: repeated HRI trials under different planner configurations for navigation, and both controlled and noisy conditions for voice interaction.

### 3.3 Reference performance indicators captured

To support interpretation of the released data, the current version of the dataset includes a small set of *reference performance indicators* derived from the recorded trials. These indicators are not presented here as the outcome of a standalone comparative study, but rather as examples of the type of benchmarking information that can be extracted from the dataset and reused in future work.

**FIGURE 2:**
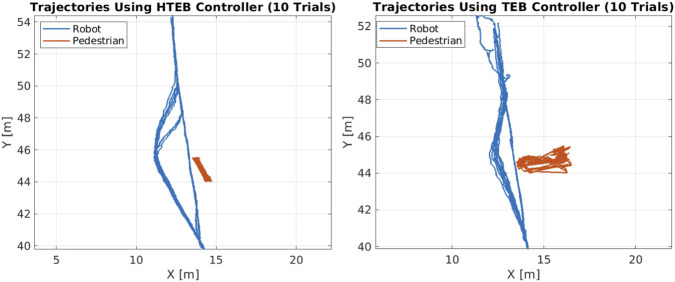
Robot-pedestrian interaction trajectories over 10 navigation trials in a head-on corridor-like scenario. Only the interaction phase is shown, and pedestrian trajectories are visible only within the robot camera field of view. Left: CoHAN-based controller, with smoother motion and larger lateral clearance. Right: baseline TEB controller, with tighter passing behaviour. Blue curves denote robot trajectories and red curves denote pedestrian trajectories.

For the *socially aware navigation* subset, the dataset contains paired runs collected with two local planning configurations: a baseline TEB planner and a CoHAN-based configuration built around HATEB. In this release, the recorded trials show zero collisions and zero close-contact events for both planner configurations, while the average minimum pedestrian clearance captured in the dataset increases from approximately 0.89 m for the TEB baseline to 1.40 m for the CoHAN-based configuration. These values illustrate the type of planner-level comparison enabled by the dataset in a heavy-duty outdoor robot setting. Examples of the experimental configurations from which these indicators are derived are shown in Figure 2.

In addition to these discrete safety indicators, the dataset also supports the computation of more continuous and predictive measures of interaction quality. In particular, two complementary indicators are included: the *Panic Cost*, which models the level of discomfort induced by robot motion in proximity to humans, and the *Time-to-Collision (TTC)*, which estimates the temporal margin before a potential collision under current motion conditions (Singamaneni et al., 2023).

- Minimum pedestrian clearance (mean ± standard deviation)
  ○ TEB: 0.895±0.120 m
  ○ CoHAN: 1.399±0.320 m
- Panic Cost: (mean ± standard deviation)
  ○ TEB: 0.118±0.008 $s^{-1}$

  ○ CoHAN: 0.092±0.008 $s^{-1}$

- TTC: median (IQR)
  ○ TEB: 15.530 s (14.762–16.776)
  ○ CoHAN: 27.338 s (23.153–30.372)

The observed differences were consistent across trials. In all 10 paired runs, the CoHAN-based planner maintained a larger minimum clearance than the corresponding TEB run, indicating that the reported improvement is not driven by isolated outliers.

For the *voice-based HRI* subset, the dataset includes command-recognition outcomes obtained under both controlled and construction-like acoustic conditions. The corresponding reference indicators include *command recognition accuracy* under each condition. In the current dataset, the recorded values are 93% for indoor playback, 73% for outdoor playback under loader-engine noise, 100% for live speech at 2 m, and 20% for live speech at 5 m. These values characterise the acoustic variability represented in the dataset and illustrate its relevance for studying command understanding under realistic field conditions.

The low accuracy observed at 5 m should be interpreted as a failure mode included in the dataset and highlights the challenges of speech interaction around heavy machinery.

Beyond overall accuracy, the dataset also allows finer-grained analysis of the interaction pipeline, including failure modes such as Automatic Speech Recognition (ASR) timeouts, substitution errors, and distance-related degradation effects. In particular, the sharp performance drop observed at increased operator distance highlights the impact of signal-to-noise ratio and microphone placement, providing a basis for evaluating improvements in audio preprocessing and interaction design.

### 3.4 Coverage and limitations

The released dataset is designed to support the evaluation of socially aware navigation and voice-based human–robot interaction in construction-like environments. While it provides a rich and multi-modal representation of real-world operation, its scope is intentionally bounded by the experimental design and platform constraints. This section outlines both the coverage of the dataset and its inherent limitations, to guide appropriate interpretation and reuse.

#### 3.4.1 Dataset coverage

The dataset captures robot operation in a controlled outdoor construction testbed. The navigation scenario consists of a corridor-like configuration, where spatial constraints naturally induce interaction between the robot and a pedestrian. This setup reflects common real-world situations such as narrow passages between equipment, materials, or temporary barriers. It includes a comprehensive set of synchronised data streams covering:

- Perception: stereo vision, LiDAR point clouds, and pedestrian detections;
- Localisation: LiDAR-inertial odometry, GNSS-RTK, and IMU measurements;
- Planning and control: global paths, local trajectories, and predicted motion;
- Interaction: voice commands, ASR outputs, and NLU dialogue acts;
- Evaluation metrics: distance measures, event indicators, and derived KPIs.

This multi-modal coverage enables end-to-end analysis of the autonomy stack, from raw sensor input to high-level decision-making.

#### 3.4.2 Dataset limitations

The dataset focuses on a single interaction topology, namely, head-on encounters with one pedestrian. As a result:

- Multi-agent scenarios (e.g., multiple pedestrians) are not represented;
- Complex interaction patterns (e.g., crossing flows, group behaviour) are absent; and
- Environmental variability is limited to a fixed spatial layout.

While this controlled setup is suitable for isolating planner behaviour, it may not fully capture the diversity of real-world deployments.

Furthermore, pedestrian perception relies primarily on onboard vision systems, which introduces several limitations, namely, Field-Of-View dependency, occlusion sensitivity and range limitation. Consequently, interaction data are inherently bounded to the observable region, which may truncate trajectories and affect temporal analysis.

Although the system leverages high-accuracy GNSS-RTK and LiDAR-based odometry, the dataset does not include an external motion-capture ground truth system. Pedestrian “ground truth” is derived from the perception pipeline and robot localisation is subject to residual drift and sensor noise. This may introduce small errors in absolute positioning, particularly relevant for fine-grained metric computation.

The current voice-interaction pipeline should be considered reliable primarily for close-range operation under the tested conditions. While recognition accuracy reached 100% at 2 m, performance degraded significantly at 5 m, where only 20% of commands were successfully recognised. Analysis of the recorded transcripts indicates that most failures originated at the ASR stage, appearing either as low-confidence substitutions or timeouts caused by reduced signal-to-noise ratio. Consequently, the current dataset should be interpreted as representing both successful and failure cases of voice interaction in construction-like environments rather than as evidence of robust long-range speech interaction.

The current voice-interaction pipeline should be considered reliable primarily for close-range operation under the tested conditions. While recognition accuracy reached 100% at 2 m, performance degraded significantly at 5 m, where only 20% of commands were successfully recognised. Analysis of the recorded transcripts indicates that most failures originated at the ASR stage, appearing either as low-confidence substitutions or timeouts caused by reduced signal-to-noise ratio. Consequently, the current dataset should be interpreted as representing both successful and failure cases of voice interaction in construction-like environments rather than as evidence of robust long-range speech interaction.

In addition, the dataset includes a limited command vocabulary and the interaction is constrained to single-command utterances. These factors limit generalisation to more complex dialogue scenarios or large-vocabulary speech recognition tasks.

## Data Availability

The datasets presented in this study can be found in online repositories. The names of the repository/repositories and accession number(s) can be found below: https://zenodo.org/records/19607695

## Footnotes

1
https://www.eurobin-project.eu/

2
https://github.com/LAAS-HRI/CoHAN2.0

3
https://github.com/socrob/nlu_rule_based
